# A higher monocyte-to-lymphocyte ratio is correlated with impaired glomerular function and adverse cardiac remodeling in elderly patients with atrial fibrillation: a retrospective study

**DOI:** 10.3389/fcvm.2026.1745544

**Published:** 2026-07-07

**Authors:** Xinrui Chen, Gang Li, Henri Mustonen, Jari A. Laukkanen, Linping Wei, Yufeng Li

**Affiliations:** 1 Division of Cardiology, Department of Geriatrics, Laboratory of Research and Translation for Geriatric Diseases, The First Affiliated Hospital of Chongqing Medical University, Chongqing, China; 2 Faculty of Medicine, University of Helsinki, Helsinki, Finland; 3 Department of Medicine, Institute of Clinical Medicine, University of Eastern Finland, Kuopio, Finland; 4 Institute of Public Health and Clinical Nutrition, University of Eastern Finland, Kuopio, Finland; 5 Department of Medicine, Wellbeing Services County of Central Finland, Jyväskylä, Finland

**Keywords:** atrial fibrillation, cardiac remodeling, elderly, glomerular filtration, dysfunction, monocyte/lymphocyte ratio

## Abstract

**Background:**

Atrial fibrillation (AF) is associated with increased cardiovascular mortality. Cardiac remodeling is a non-negligible pathological mechanism for the elevated risk of death in patients with AF. In addition, an elevated systemic inflammatory response is associated with adverse cardiac remodeling and increased mortality. However, it remains incompletely understood whether inflammation markers, such as the monocyte/lymphocyte ratio (MLR), are associated with adverse cardiac remodeling and clinical biochemical indexes in patients with AF. Therefore, this study investigated the association between MLR and clinical biochemical indexes and cardiac remodeling in elderly patients with AF.

**Methods:**

In a single medical care center, a total of 1,154 elderly Chinese hospitalized patients (aged ≥ 65 years) with AF were collected retrospectively. The patients were divided into low (≤ 0.293), moderate (> 0.293 to ≤ 0.460), and high (> 0.460) MLR groups according to the MLR tertiles. A regression analysis of MLR (> 0.460) with clinical biochemical indexes and echocardiographic parameters was conducted.

**Results:**

The results revealed that high MLR (> 0.460) was independently associated with male sex, decreased estimated glomerular filtration rate (eGFR), lower plasma albumin level, cardiac ventricular dilatation, and cardiac dysfunction (all *P* < 0.05).

**Conclusion:**

High MLR was linked to male sex, decreased eGFR, lower plasma albumin level, and adverse cardiac remodeling in elderly patients with AF.

## Introduction

Atrial fibrillation (AF) is a common supraventricular arrhythmia in clinical practice, especially in the elderly population. Increased stroke risk has been confirmed in patients with AF (1). The death rate resulting from thromboembolism-related events in elderly patients with AF has been shown to decrease after treatment with anticoagulant medication. However, the risk of death from cardiovascular events remains elevated in patients with AF, even when treated with appropriate anticoagulant medication (2). Serious arrhythmias were usually first considered to be responsible for increased cardiovascular mortality in patients with AF (1). Furthermore, adverse cardiac remodeling may be an important pathological substrate for higher cardiovascular mortality in patients with AF (3, 4). Surveys have suggested that chronic inflammation is associated with increased incidence of AF in patients with adverse cardiac structural abnormalities (5, 6). Elevated inflammatory markers, such as C-reactive protein and interleukin-6, have been reported to be associated with adverse cardiac remodeling and increased cardiovascular death in patients with AF (7, 8). Moreover, an additional easily available inflammatory marker, the monocyte/lymphocyte ratio (MLR), has been reported to be associated with increased cardiovascular mortality (9). However, it remains unclear whether MLR is associated with adverse cardiac remodeling. Therefore, this study investigated the association between MLR and cardiac remodeling and related clinical biochemical indexes in elderly patients with AF.

## Materials and methods

### Study design and participants

A cross-sectional study was conducted, in which 1,154 elderly Chinese hospitalized patients (aged ≥ 65 years) with non-valvular AF [persistent AF (*n* = 596); long-term persistent AF (*n* = 442); permanent AF (*n* = 116)] were collected retrospectively through a review of medical records from 2014 to 2021 in our inpatient department. All the patients had undergone electrocardiographic examinations, color Doppler echocardiography, and biochemical tests (10). This research was conducted in accordance with the Declaration of Helsinki and approved by the ethics committee of the First Affiliated Hospital of Chongqing Medical University (No. 2022-K390, 1 September 2022). The ethics committee granted a waiver of informed consent due to the anonymous nature of the retrospectively acquired clinical data.

White blood cell (WBC) count in peripheral blood was assessed using a Sysmex XN-1000 Analyzer (Sysmex Corporation, Kobe, Japan). The MLR was then calculated manually. WBC and MLR were measured three times on average within the first week of hospitalization for each patient, respectively. The patients with AF were divided into the low MLR group (the ratio ≤ 0.293, *n* = 382), moderate MLR group (the ratio > 0.293 to ≤ 0.460, *n* = 392), and high MLR group (the ratio > 0.460, *n* = 380), according to the MLR tertiles.

Patients were excluded if they had acute stroke, myocardial infarction (MI), gout attack, hypertrophy, restrictive or dilated cardiomyopathy, valvular or congenital heart diseases, chronic obstructive pulmonary disease, infection, autoimmune diseases, glomerulonephritis, and/or malignant tumor or if they had not undergone electrocardiographic and echocardiographic examinations or the necessary blood biochemical tests.

### Diagnostic criteria for comorbidities

Type 2 diabetes mellitus (DM) was confirmed if the patient had previously diagnosed type 2 DM in which insulin or oral glucose-lowering medication had been administered or if their fasting plasma glucose was ≥ 7.0 mmol/L and/or their plasma glucose was ≥ 11.1 mmol/L 2 h after an oral 75 g glucose-loaded tolerance test, while their plasma insulin level was normal or elevated. Patients with diabetic ketoacidosis or other types of DM or in a hyperosmolar state were excluded (11).

Primary hypertension was confirmed as follows: after a 10 min rest and two blood pressure (BP) measurements, the patient’s average arterial systolic BP was ≥140 mmHg and/or diastolic BP was ≥90 mmHg on different days without taking antihypertensive drugs; or if hypertension had been diagnosed previously and antihypertensive medications had been used regularly. Patients with secondary hypertension were excluded (12).

Gout was defined as urate crystals in the joints with gouty stones, arthralgia, and arthritis (13).

Coronary heart disease (CHD) was diagnosed if the patients had at least one main branch with atherosclerotic lumen stenosis (≥ 50%) in the coronary artery confirmed by angiography, if the patient had confirmed MI, or they had a history of coronary stent placement (14). The severity of coronary artery lesions was assessed using the Gensini score (15).

Stroke was diagnosed based on positive brain imaging evidence from electronic computed tomography (CT) or magnetic resonance imaging (MRI), or previously diagnosed stroke with or without a previous history of hemiparesis (16).

### Detection of plasma lipids, glucose, glycated hemoglobin, and high-sensitivity C-reactive protein

Plasma glucose was measured using the hexokinase colorimetric method, and glycosylated hemoglobin (HbA1c) was measured using high-performance liquid chromatography. Triglycerides and total cholesterol were detected using the enzyme colorimetric method, and high-density lipoprotein cholesterol (HDL-C) was determined using the homogeneous enzyme colorimetric method. Low-density lipoprotein cholesterol (LDL-C) was calculated using the Friedewald formula (17). Albumin was measured using the bromocresol green method. D-dimer was determined using immunoturbidimetry (18). High-sensitivity C-reactive protein (hs-CRP) was determined using an immunoturbidimetric assay.

### Determination of glomerular filtration rate and body mass index

For men, if the serum creatinine (Scr) was > 80 µmol/L, the estimated glomerular filtration rate (eGFR) = 141 × (Scr µmol/L/88.4/0.9)^−1.209^ × 0.993^Age (years)^ and if Scr was ≤ 80 µmol/L, eGFR = 141 × (Scr µmol/L/88.4/0.9)^−0.411^ × 0.993^Age (years)^. For women, if Scr was > 62 µmol/L, eGFR = 144 × (Scr µmol/L/88.4/0.7)^−1.209^ × 0.993^Age (years)^ and if Scr was ≤ 62 µmol/L, eGFR = 144 × (Scr µmol/L/88.4/0.7)^−0.329^ × 0.993^Age (years)^ (19, 20). Body mass index (BMI) was calculated as body weight (kg)/[height (m)]^2^ (21).

### Measurement of cardiac structure and function

Using a GE Vivid7 full digital color Doppler ultrasound diagnostic instrument (GE HealthCare, UK), transthoracic two-dimensional M-mode echocardiography was conducted to determine (22) the following: right atrial internal diameter (RAD), right ventricular internal diameter (RVD), left atrial internal diameter (LAD), interventricular septal thickness (IVST), left ventricular posterior wall thickness (LVPWT), left ventricular end-systolic internal diameter (LVESD), left ventricular end-diastolic internal diameter (LVEDD), pulmonary artery pressure (PAP), and left ventricular ejection fraction (LVEF) (23–25).

### Statistical analysis

The Statistical Package for the Social Sciences (SPSS) 26.0 software (IBM, Chicago, IL, USA) was used for the statistical analysis. The cutoff tertile MLR values used in this study were calculated using SPSS, according to the MLR values of the elderly patients. Continuous data are expressed as mean ± standard deviation. The between-group comparisons in the normally distributed continuous data were made using *t*-tests. If the continuous data did not follow a normal distribution, the Mann–Whitney *U* test was used for the between-group comparisons. Counting data are expressed as a percentage (%), and the chi-square test was conducted. The Pearson or Spearman correlation analysis was performed in the univariate analysis. Dichotomized logistic regression analysis was used in the multifactorial analysis. Because the hs-CRP, neutrophil, and WBC values were collinear with MLR, these parameters were not included in the regression analysis. A two-tailed value of *P* < 0.05 was considered statistically significant.

## Results

### Clinical characteristics according to tertile MLR

The clinical characteristics of subjects according to MLR tertile are shown in Table 1. Compared with the low MLR group (≤0.293), the moderate MLR (>0.293 to ≤0.460) group had older age; higher rates of smoking, alcohol consumption, and gout; higher D-dimer, hs-CRP, and neutrophil levels (all *P* < 0.05, Table 1); and lower BMI, diastolic BP, TG, LDL-C, albumin, and eGFR (all *P* < 0.05, Table 1). Moreover, compared with the low MLR group, the high MLR (>0.460) group had older age; higher rates of male sex, smoking, alcohol consumption, and gout; higher D-dimer, hs-CRP, neutrophil, and WBC levels (all *P* < 0.05, Table 1); and lower BMI, diastolic BP, TG, LDL-C, HDL-C, albumin, and eGFR (all *P* < 0.05, Table 1). Compared with the moderate MLR group (>0.293 to ≤0.460), the high MLR group (>0.460) had older age; higher rates of smoking and alcohol consumption; higher hs-CRP, neutrophil, and WBC levels (all *P* < 0.05, Table 1); and lower HDL-C, albumin, and eGFR (all *P* < 0.05, Table 1).

**Table 1:** Comparison of clinical characteristics among tertile MLR groups.

Clinical characteristics	Low MLR	Moderate MLR	High MLR	*r*	*P*
	MLR ≤ 0.293	0.293 < MLR ≤ 0.460	MLR > 0.460		
	*n* = 382	*n* = 392	*n* = 380		
MLR	0.217 ± 0.05	0.36 ± 0.05^a^	0.75 ± 0.38^a,b^	1	＜0.001
Male [*n* (%)]	132 (34.6)	158 (40.3)	199 (52.4)^a^	0.146	＜0.001
Age (year)	76.93 ± 7.30	78.03 ± 6.71^a^	80.22 ± 7.26^a,b^	0.185	＜0.001
BMI (kg/m^2^)	24.51 ± 3.90	23.95 ± 3.97^a^	23.45 ± 4.26^a^	−0.112	0.001
Smoking [*n* (%)]	73 (19.1)	96 (24.5)^a^	104 (27.4)^a,b^	0.079	0.024
Drinking [*n* (%)]	45 (11.8)	63 (16.1)^a^	71 (18.7)^a,b^	0.078	0.028
Systolic BP (mmHg)	132.02 ± 20.30	131.90 ± 21.49	130.76 ± 23.95	−0.023	0.702
Diastolic BP (mmHg)	80.50 ± 14.66	78.18 ± 15.13^a^	76.09 ± 15.69^a^	−0.117	＜0.001
Persistent AF [*n* (%)]	193 (50.5)	205 (52.3)	198 (52.1)	0.013	0.869
Long-term persistent AF [*n* (%)]	152 (39.8)	142 (36.2)	148 (38.9)	−0.007	0.569
Permanent AF [*n* (%)]	37 (9.7)	45 (11.5)	34 (8.9)	−0.010	0.492
Type 2 DM [*n* (%)]	120 (31.4)	125 (31.9)	126 (33.2)	0.015	0.874
DM duration (years)	2.64 ± 5.93	3.04 ± 6.81	3.36 ± 7.07	0.044	0.329
Hypertension [*n* (%)]	278 (72.8)	290 (74.0)	264 (69.5)	−0.030	0.355
Hypertension duration (years)	9.87 ± 11.49	11.16 ± 12.26	11.16 ± 12.30	0.044	0.215
Gout [*n* (%)]	7 (1.8)	22 (5.6)^a^	22 (5.8)^a^	0.078	0.010
Gout duration (years)	0.08 ± 0.92	0.38 ± 2.71	0.34 ± 2.27	0.050	0.103
Coronary heart disease [*n* (%)]	166 (43.5)	167 (42.6)	185 (48.7)	0.043	0.187
Gensini score	3.33 ± 9.48	4.01 ± 11.23	3.97 ± 11.61	0.024	0.621
Stroke [*n* (%)]	78 (20.4)	98 (25.0)	99 (26.1)	0.054	0.151
HbA1c (%)	6.37 ± 1.17	6.35 ± 1.17	6.46 ± 1.44	0.028	0.498
Triglyceride (mmol/L)	1.30 ± 0.73	1.11 ± 0.75^a^	1.07 ± 0.65^a^	−0.131	＜0.001
LDL-C (mmol/L)	2.24 ± 0.81	1.98 ± 0.75^a^	1.90 ± 0.72^a^	−0.181	＜0.001
HLD-C (mmol/L)	1.23 ± 0.38	1.22 ± 0.37	1.13 ± 0.36^a,b^	−0.114	＜0.001
D-dimer (mmol/L)	0.90 ± 1.54	1.29 ± 2.40^a^	1.47 ± 1.94^a^	0.113	0.001
Albumin (g/L)	40.97 ± 4.43	39.82 ± 4.61^a^	37.57 ± 4.69^a,b^	−0.289	＜0.001
eGFR (mL/min/1.73m^2^)	67.76 ± 19.19	63.27 ± 20.31^a^	58.92 ± 24.56^a,b^	−0.163	＜0.001
hs-CRP (mg/L)	3.21 ± 4.93	4.97 ± 6.20^a^	9,12 ± 7.60^a,b^	0.354	＜0.001
Monocytes(×10^9^/L)	0.37 ± 0.13	0.47 ± 0.14^a^	0.60 ± 0.21^a,b^	0.545	＜0.001
Lymphocytes(×10^9^/L)	1.72 ± 0.59	1.30 ± 0.40^a^	0.89 ± 0.35^a,b^	−0.674	＜0.001
Neutrophils(×10^9^/L)	3.83 ± 1.25	4.05 ± 1.30^a^	4.67 ± 1.59^a,b^	0.445	＜0.001
White blood cells(×10^9^/L)	6.07 ± 1.53	5.97 ± 1.55	6.30 ± 1.73^a,b^	0.059	0.018

a *P* < 0.05 vs. low MLR group.

b *P* < 0.05 vs. moderate MLR Group.

Values presented as mean ± SD or *n* (%). MLR, monocyte/lymphocyte ratio; BMI, body mass index; DM, diabetes mellitus; BP, blood pressure; HbA1c, glycated hemoglobin; LDL-C, low-density lipoprotein cholesterol; HDL-C, high-density lipoprotein cholesterol; eGFR, estimated glomerular filtration rate; hs-CRP, high-sensitivity C-reactive protein.

The univariate correlation analysis showed that MLR was positively correlated with age; the prevalence of male sex, smoking, alcohol consumption, and gout; and D-dimer, hs-CRP, neutrophil, and WBC levels (all *P* < 0.05, Table 1). Furthermore, it was negatively correlated with BMI, diastolic BP, TG, LDL-C, HDL-C, albumin, and eGFR (all *P* < 0.05, Table 1).

Medication data in Table 2.

**Table 2:** Comparison of medication data among tertile MLR groups.

Medication	Low MLR group	Moderate MLR Group	High MLR group	*r*	*P*
	MLR≤0.293	0.293 < MLR≤0.460	MLR > 0.460		
	*n* = 382	*n* = 392	*n* = 380		
Nitrates [*n* (%)]	79 (19.9)	81 (20.7)	85 (22.4)	0.025	0.693
Trimetazidine [*n* (%)]	70 (18.3)	75 (19.1)	73 (19.2)	0.025	0.941
Warfarin [*n* (%)]	117 (30.6)	110 (28.1)	104 (27.4)	−0.029	0.576
Low molecular weight heparin [*n* (%)]	82 (21.5)	104 (26.5)^a^	114 (30.0)^a^^,b^	0.079	0.026
Xaban drugs [*n* (%)]	122 (31.9)	146 (37.2)	125 (32.9)	0.008	0.251
Dabigatran etexilate [*n* (%)]	85 (22.3)	81 (20.7)^a^	58 (15.3)^a^^,b^	−0.072	0.037
Deslanoside Injection [*n* (%)]	34 (8.9)	43 (11.0)	63 (16.3)^a^^,b^	0.093	0.005
Digoxin [*n* (%)]	50 (13.1)	48 (12.2)^a^	71 (18.2)^a^^,b^	0.064	0.023
Amiodarone [*n* (%)]	31 (8.1)	19 (4.8)^a^	12 (3.2)^a^^,b^	−0.089	0.008
Aspirin [*n* (%)]	83 (21.7)	69 (17.6)	84 (16.8)	−0.051	0.176
Clopidogrel [*n* (%)]	128 (33.5)	143 (36.5)	135 (35.5)	0.017	0.680
Metformin [*n* (%)]	65 (17.0)	63 (16.1)	42 (11.1)^a^^,b^	−0.068	0.044
Sulfonylurea drugs [*n* (%)]	17 (4.5)	18 (4.6)	15 (3.9)	−0.010	0.918
Meglitinides [*n* (%)]	11 (2.9)	16 (4.1)	11 (2.9)	0.001	0.573
Acarbose [*n* (%)]	46 (12.0)	43 (11.0)^a^	25 (6.6)^a^^,^^b^	−0.074	0.027
Thiazolidinediones [*n* (%)]	1 (0.3)	1 (0.3)	2 (0.5)	0.018	0.701
DPP-4 inhibitors [*n* (%)]	3 (0.8)	12 (3.1)	6 (1.6)	0.024	0.050
GLP-1 agonists [*n* (%)]	1 (0.3)	1 (0.3)	2 (0.5)	0.018	0.701
SGLT-2 inhibitors [*n* (%)]	23 (6.0)	28 (7.1)	29 (7.6)	0.026	0.673
Insulin [*n* (%)]	46 (12.0)	48 (12.2)	56 (14.7)	0.022	0.475
ACEI/ARB [*n* (%)]	255 (66.8)	260 (66.3)	244 (64.2)	−0.022	0.732
CCB [*n* (%)]	118 (30.9)	113 (28.8)	118 (31.1)	0.001	0.760
*β*-Blockers [*n* (%)]	244 (63.9)	261 (66.6)	258 (67.9)	0.035	0.490
Furosemide [*n* (%)]	186 (48.7)	234 (59.7)^a^	284 (74.7)^a^^,b^	0.217	<0.001
Thiazide diuretic [*n* (%)]	39 (10.2)	40 (10.2	31 (8.2)	−0.028	0.546
Spironolactone [*n* (%)]	147 (38.5)	160 (40.8)^a^	180 (47.4)^a^^,b^	0.073	0.036
Sodium bicarbonate [*n* (%)]	30 (7.9)	39 (9.9)	66 (17.4)^a^^,b^	0.120	<0.001
Statins [*n* (%)]	298 (78.0)	290 (74.0)	275 (72.4)	−0.053	0.181
Fibrates [*n* (%)]	1 (0.3)	1 (0.3)	0 (0)	−0.026	1.000
Compound *α*-ketoacid tablets [*n* (%)]	6 (1.6)	9 (2.3)	28 (7.4)^a^^,b^	0.124	<0.001
Radiofrequency ablation [*n* (%)]	40(10.5)	39(10.0)	16(4.2)^a^^,b^	−0.093	0.002

a *P* < 0.05 vs. low MLR group;

b *P* < 0.05 vs. moderate MLR group.

Values presented as mean ± SD. MLR, Monocyte/lymphocyte ratio; ACEI, Angiotensin-Converting Enzyme Inhibitors; ARB, Angiotensin II Receptor Blockers; CCB, Calcium Channel Blockers.

### Cardiac remodeling according to MLR tertile

Compared with the low MLR group, RAD, RVD, and LAD were larger in the moderate MLR group (all *P* < 0.05, Table 3); RAD, RVD, LAD, LVESD, and LVEDD were larger, PAP was higher, and LVEF was lower in the high MLR group (all *P* < 0.05, Table 3). Compared with the moderate MLR group, RAD, RVD, LVESD, and LVEDD were larger, PAP was higher, and LVEF was lower in the high MLR group (all *P* < 0.05, Table 3).

**Table 3:** Comparison of echocardiographic parameters among tertile MLR groups.

Echocardiographic parameters	Low MLR group	Moderate MLR Group	High MLR group	*r*	*P*
	MLR ≤ 0.293	0.293 < MLR ≤ 0.460	MLR > 0.460		
	*n* = 382	*n* = 392	*n* = 380		
RAD (mm)	41.94 ± 5.32	43.56 ± 6.75^a^	44.75 ± 6.82^a,b^	0.178	＜0.001
RVD (mm)	20.60 ± 3.06	21.34 ± 3.44^a^	22.30 ± 4.40^a,b^	0.184	＜0.001
LAD (mm)	39.96 ± 6.09	41.09 ± 6.04^a^	41.92 ± 6.15^a^	0.129	＜0.001
IVST (mm)	10.65 ± 1.19	10.69 ± 1.39	10.68 ± 1.49	0.010	0.881
LVPWT (mm)	10.48 ± 1.13	10.52 ± 1.26	10.52 ± 1.28	0.012	0.875
LVESD (mm)	33.45 ± 6.23	33.95 ± 7.00	35.33 ± 8.08^a,b^	0.107	0.001
LVEDD (mm)	47.66 ± 6.01	48.44 ± 6.90	49.61 ± 7.94^a,b^	0.113	0.001
PAP (mmHg)	39.04 ± 10.22	40.40 ± 10.23	43.54 ± 12.26^a,b^	0.165	＜0.001
LVEF (%)	58.38 ± 8.30	58.05 ± 8.79	56.15 ± 9.79^a,b^	−0.100	0.002

a *P* < 0.05 vs. low MLR group.

b *P* < 0.05 vs. moderate MLR group.

Values presented as mean ± SD. MLR, monocyte/lymphocyte ratio; RAD, right atrial diameter; RVD, right ventricular diameter; LAD, left atrial diameter; IVST, interventricular septal thickness; LVPWT, left ventricular posterior wall thickness; LVESD, left ventricular end-systolic diameter; LVEDD, left ventricular end-diastolic diameter; PAP, pulmonary artery pressure; LVEF, left ventricular ejection fraction.

The univariate correlation analysis revealed that MLR was positively correlated with RAD, RVD, LAD, LVESD, LVEDD, and PAP (all *P* < 0.05, Table 3), and negatively correlated with LVEF (*P* < 0.05, Table 3).

### Multivariate logistic regression analysis of MLR, clinical characteristics, and echocardiographic parameters

**Figure 1:**
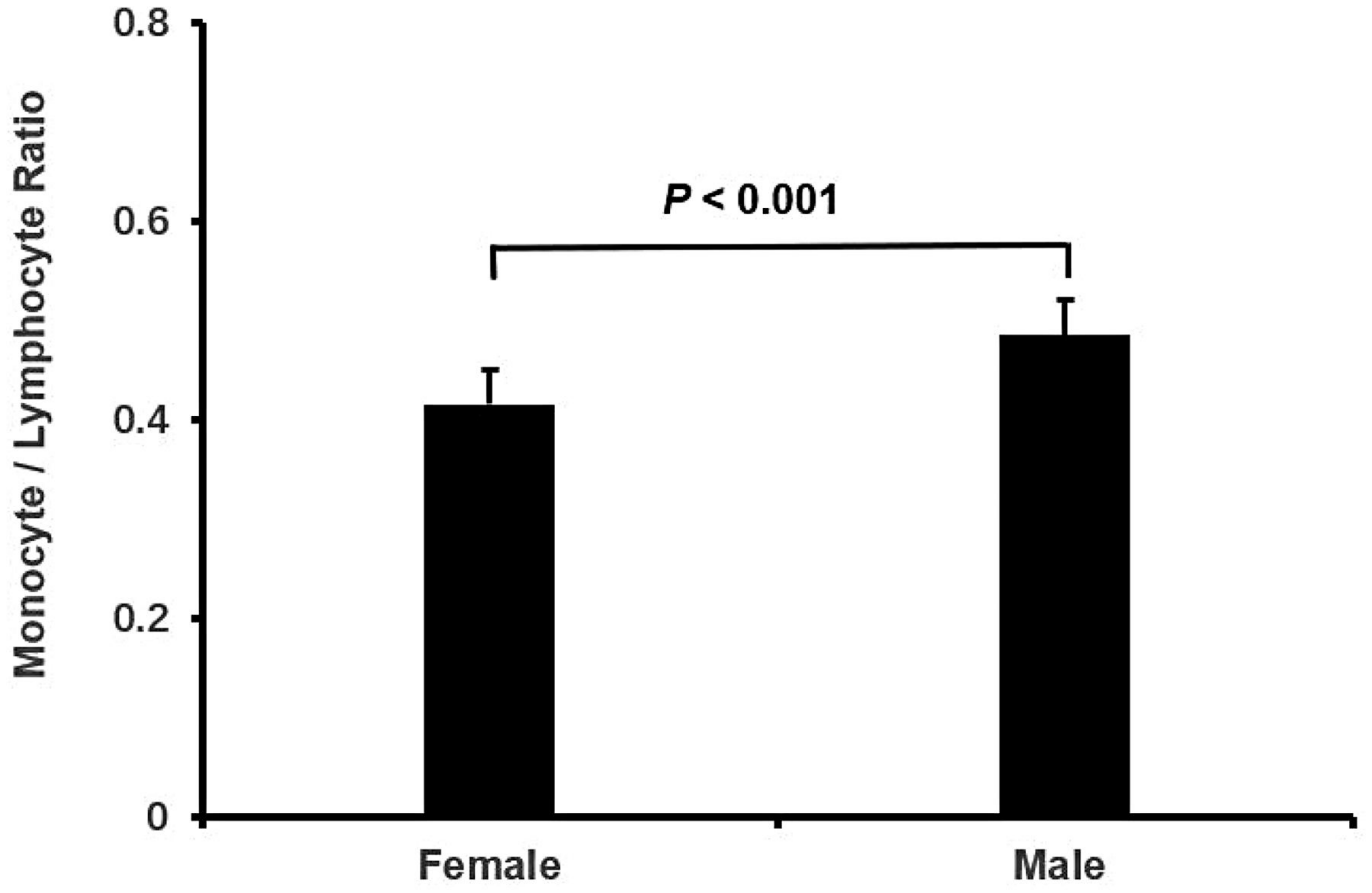
The monocyte/lymphocyte ratios in men and women.

**Figure 2:**
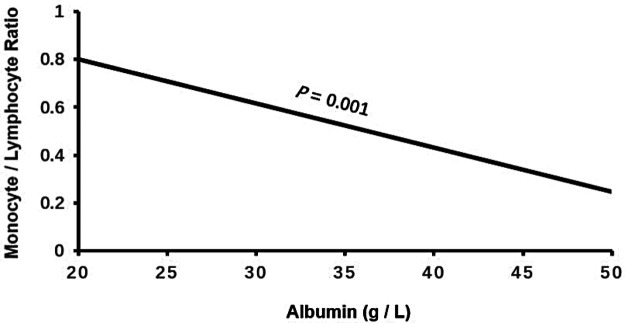
The association between monocyte/lymphocyte ratio and plasma albumin.

**Figure 3:**
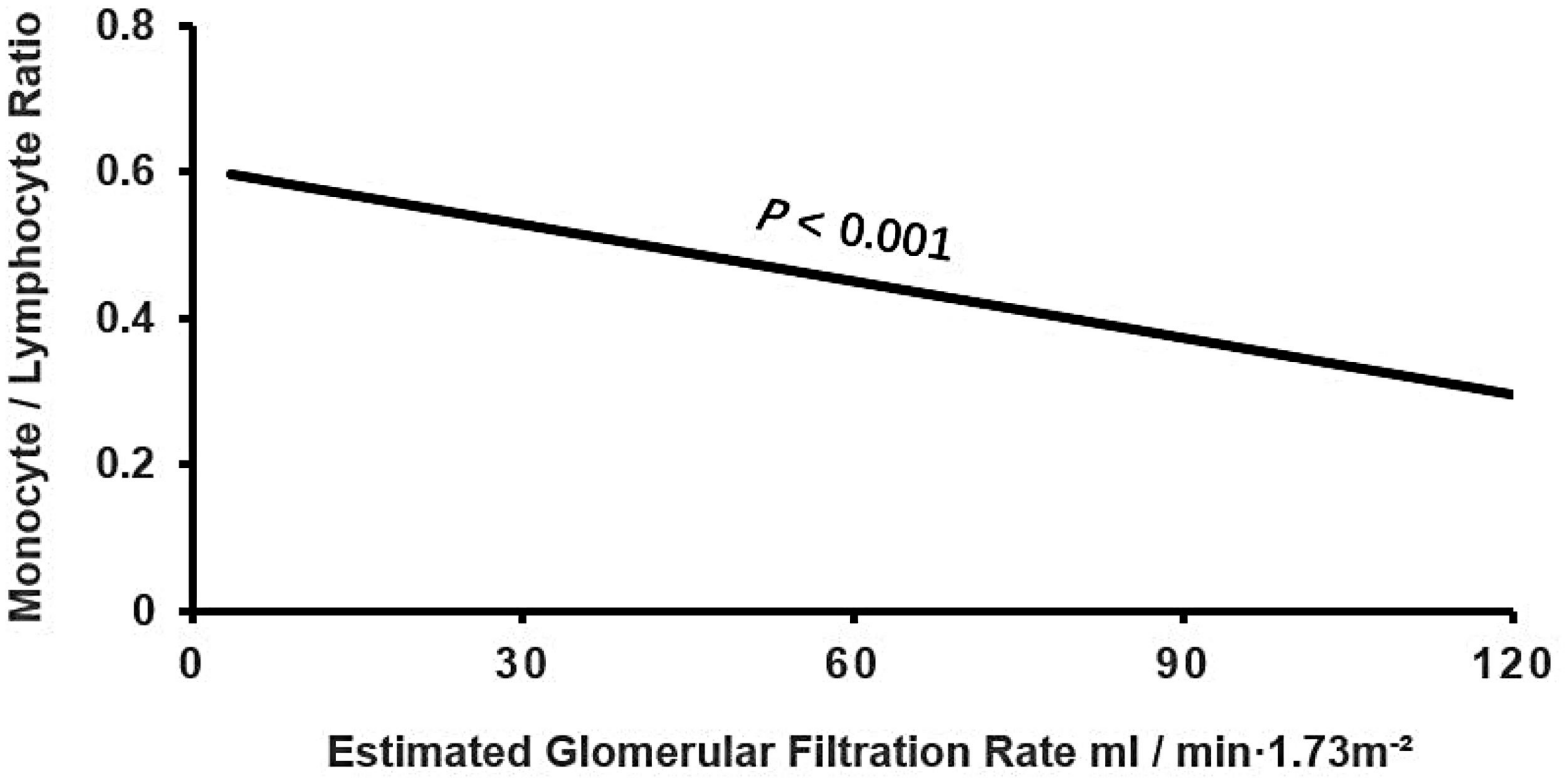
The association between monocyte/lymphocyte ratio and estimated glomerular filtration rate.

**Figure 4:**
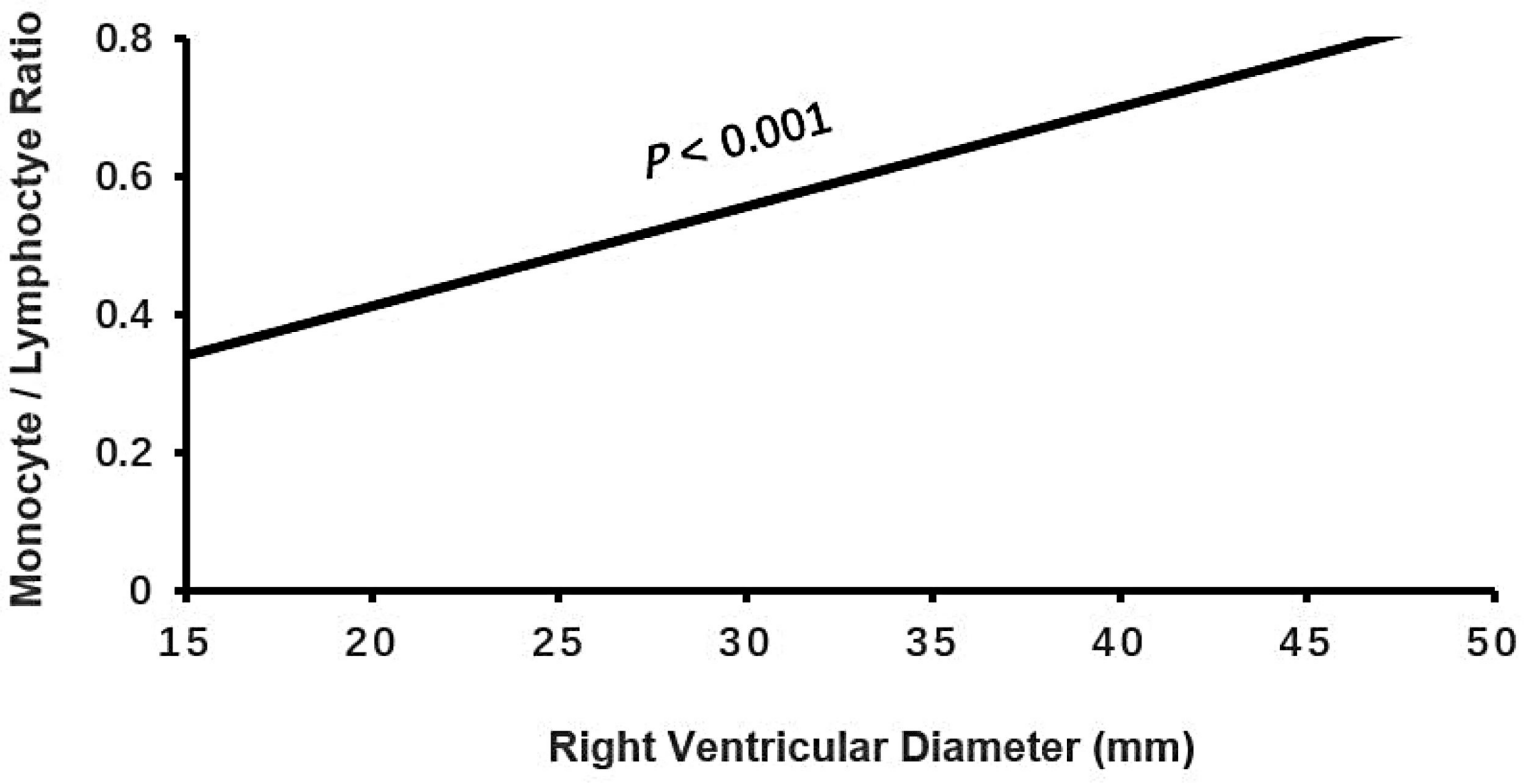
The association between monocyte/lymphocyte ratio and right ventricular diameter.

**Figure 5:**
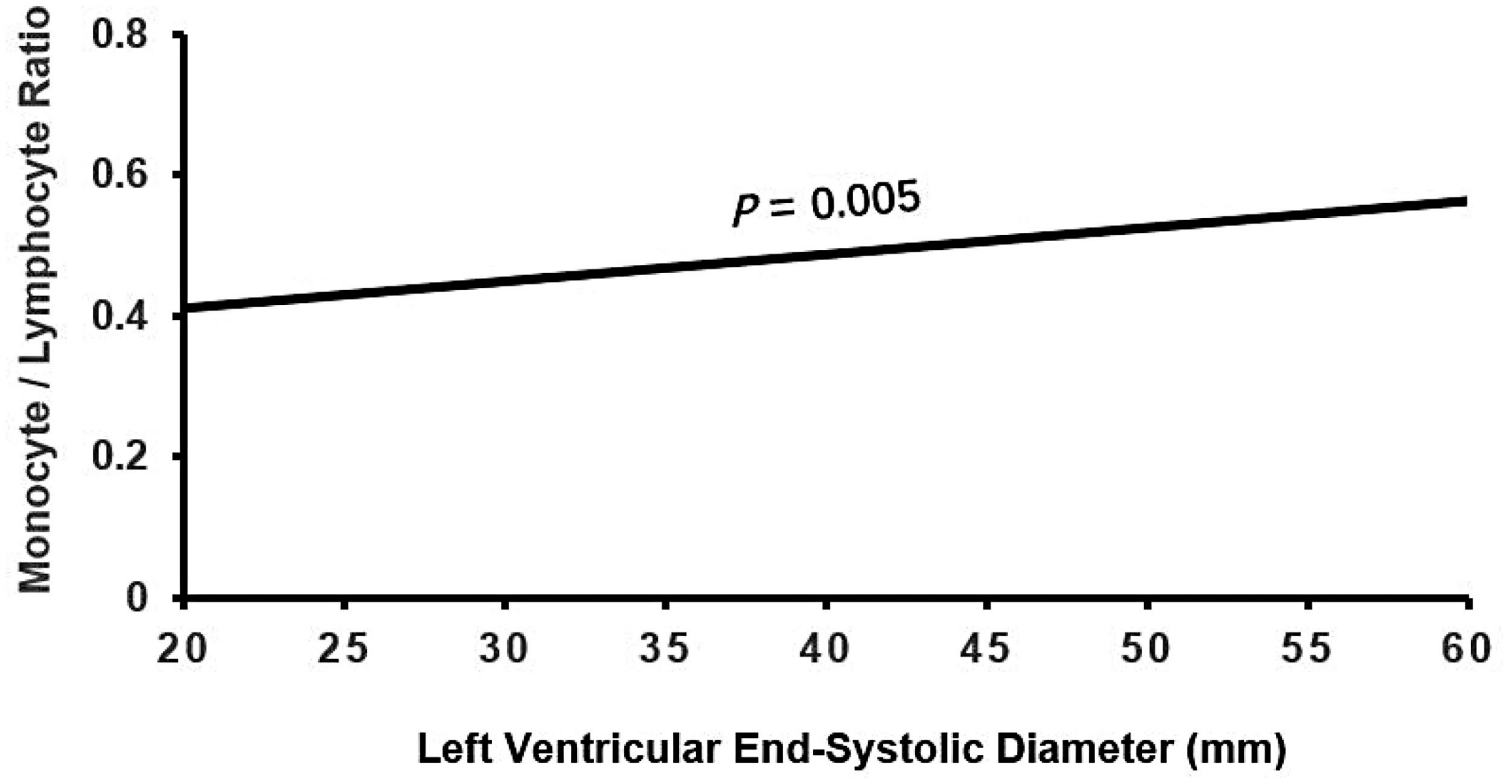
The association between monocyte/lymphocyte ratio and left ventricular end-systolic diameter.

**Figure 6:**
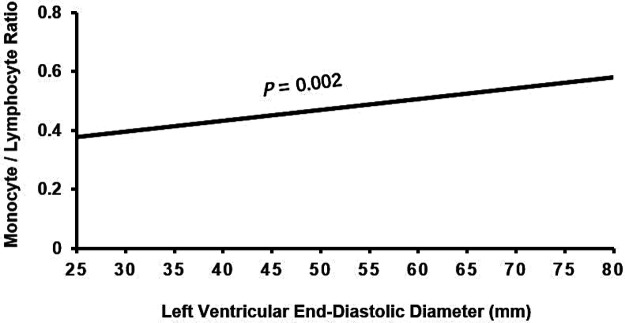
The association between monocyte/lymphocyte ratio and left ventricular end-diastolic diameter.

**Figure 7:**
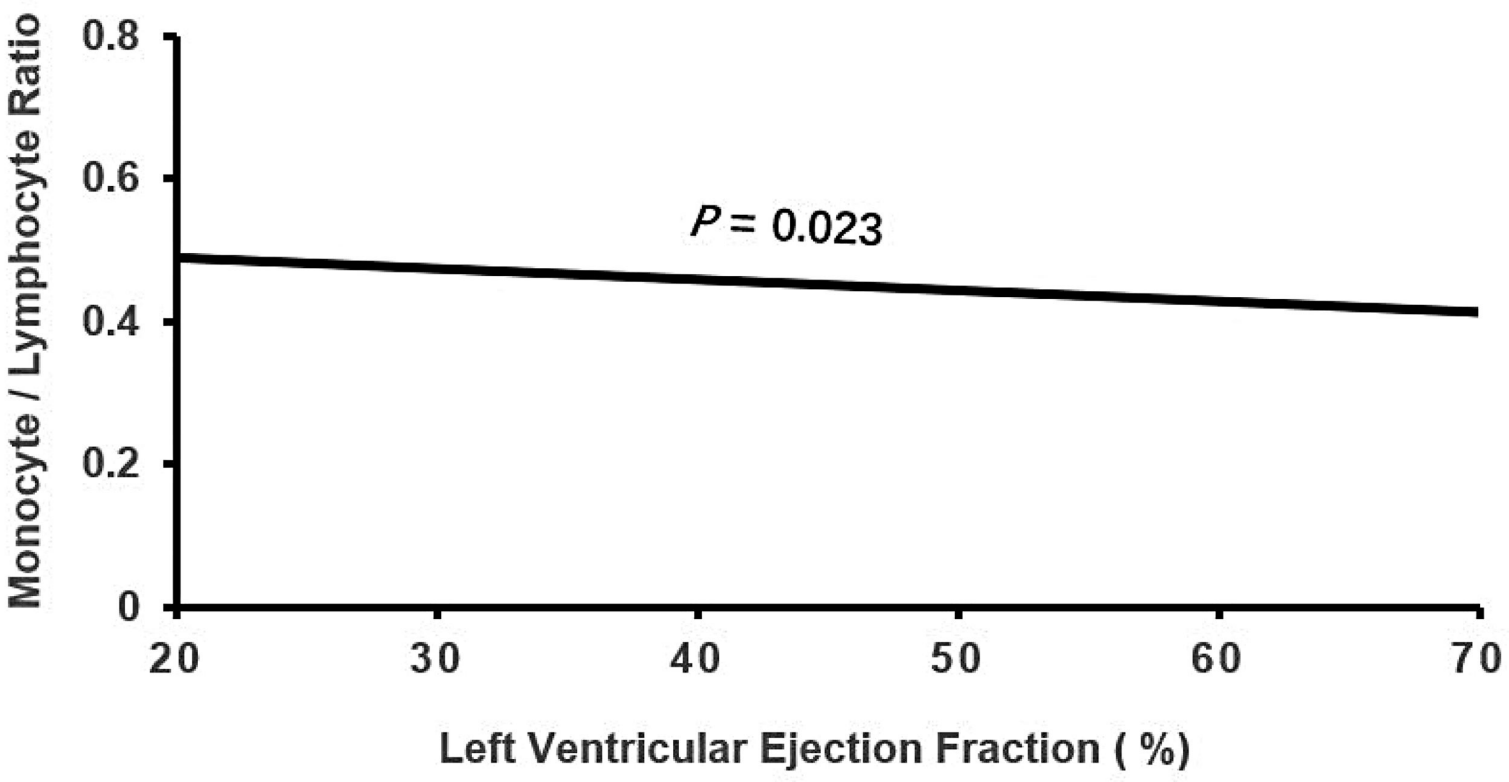
The association between monocyte/lymphocyte ratio and left ventricular ejection fraction.

Multivariate logistic binary regression analysis of clinical characteristics and echocardiographic parameters in the high MLR group (MLR > 0.460) showed that high MLR was independently associated with male sex; decreased plasma albumin level; lower eGFR; larger RVD, LVESD, and LVEDD; and lower LVEF (all *P* < 0.05, Table 4; Figures 1–7).

**Table 4:** Regression analysis of clinically echocardiographic parameters with MLR > 0.460.

Parameters	*β*	SE	Wald *χ*^2^	*P*	OR (95% CI)
Male	1.173	0.292	16.192	<0.001	3.233 (1.825–5.725)
Age	0.011	0.017	0.44	0.507	1.011 (0.978–1.046)
BMI	−0.009	0.026	0.128	0.720	0.991 (0.941–1.043)
Smoking	−0.344	0.383	0.804	0.370	0.709 (0.334–1.503)
Drinking	−0.321	0.39	0.677	0.411	0.725 (0.337–1.559)
Diastolic BP	−0.008	0.007	1.201	0.273	0.992 (0.978–1.006)
Gout	0.314	0.478	0.433	0.510	1.370 (0.537–3.494)
Triglycerides	0.174	0.211	0.684	0.408	1.190 (0.788–1.800)
LDL-C	0.063	0.155	0.163	0.686	1.065 (0.786–1.442)
HDL-C	−0.186	0.318	0.341	0.559	0.831 (0.446–1.549)
D-dimer	−0.027	0.07	0.147	0.701	0.974 (0.849–1.116)
Albumin	−0.136	0.027	25.513	<0.001	0.872 (0.827–0.92)
eGFR	−0.013	0.005	5.852	0.016	0.987 (0.977–0.998)
RAD	−0.021	0.023	0.851	0.356	0.979 (0.936–1.024)
RVD	0.105	0.036	8.486	0.004	1.110 (1.035–1.192)
LAD	−0.016	0.022	0.562	0.453	0.984 (0.943–1.026)
LVESD	−0.153	0.072	4.575	0.032	0.858 (0.746–0.987)
LVEDD	0.108	0.053	4.207	0.040	1.114 (1.005–1.234)
PAP	0.018	0.01	3.318	0.069	1.018 (0.999–1.038)
LVEF	−0.071	0.029	6.239	0.012	0.931(0.880–0.985)

MLR, monocyte/lymphocyte ratio; *β*, regression coefficient; SE, standard error; Wald, chi-square value; CI, confidence interval; OR, odds ratio; BMI, body mass index; BP, blood pressure; LDL-C, low-density lipoprotein cholesterol; HDL-C, high-density lipoprotein cholesterol; eGFR, estimated glomerular filtration rate; RAD, right atrial diameter; RVD, right ventricular diameter; LAD, left atrial diameter; LVESD, left ventricular end-systolic diameter; LVEDD, left ventricular end-diastolic diameter; PAP, pulmonary artery pressure; LVEF, left ventricular ejection fraction.

## Discussion

This study showed that increased MLR was independently associated with male sex, decreased plasma albumin level, lower eGFR, adverse cardiac remodeling, and cardiac dysfunction in elderly patients with AF.

Previous studies have suggested that dyslipidemia and obesity are associated with a high-fat diet and contribute to lipid deposition and lipotoxicity in the kidneys (26–28). Lipotoxicity can lead to insulin resistance and impede glucose utilization by kidney cells, with the energy supply from fat oxidation being forced to increase subsequently. However, this manner of energy supply can lead to the release of pro-inflammatory factors, which increase apoptosis and necrosis of endothelial and epithelial cells in glomeruli (27, 29, 30), elevate urinary albumin, decrease the plasma albumin level and eGFR (31–33), and cause renal dysfunction (34). With decreased eGFR and increased retention of plasma toxins, a suppressed appetite, decreased protein intake, and increased leakage of plasma albumin ultimately lead to hypoalbuminemia and metabolic nephropathy (35). A high MLR has been associated with low eGFR in chronic kidney disease (36). However, to our knowledge, this study is the first to demonstrate that high MLR is associated with low eGFR and decreased plasma albumin levels in elderly patients with AF. Studies have found that unhealthy lifestyle factors, such as cigarette smoking and alcohol consumption, are associated with increased systemic inflammation, especially in older men (37, 38). However, this study is the first to confirm that a high MLR was strongly associated with being male in elderly patients with AF.

Studies have confirmed that dyslipidemia, overweight, obesity, insulin resistance, hyperglycemia, type 2 DM, and hypertension are associated with the development of AF (39–43). A growing body of data from both epidemiological and clinical studies has demonstrated that epicardial fat is associated with the severity of AF. Some studies have found a relationship between epicardial fat accumulation and atrial myocardial adipocyte infiltration due to increased body fat. The Framingham Heart Study, through CT analysis, suggested that the total amount of epicardial fat is associated with AF risk. Thus, this is a potential mechanism linking overweight and obesity, the amount of epicardial fat, and AF that could potentially lead to anisotropic conduction in the atrium wall and profibrotic adipokines, culminating in fibrosis formation, remodeling, inflammation, and autonomic nervous system (ANS) dysfunction. ANS imbalance is one of the most important pathophysiological mechanisms in the genesis of AF. Positive modulation, such as losing weight, physical activity, and enhanced cardiorespiratory fitness, is crucial for improving ANS function (44, 45).

Metabolic disorders were strongly associated with adverse cardiac structural abnormalities and dysfunctional cardiac remodeling. An increase in epicardial fat may lead to structural and dysfunctional remodeling of the heart through both direct (e.g., infiltration of adipose tissue) and indirect mechanisms (e.g., myocardial inflammation and oxidative stress) (46–50). High-fat diets have been confirmed to be associated with dyslipidemia and overweight and obesity (26). The retained lipids in tissues are responsible for the development of insulin resistance, impaired glucose tolerance, and type 2 DM (51). Insulin resistance prevents cells from utilizing glucose for their energy supply and forces them to utilize fat oxidation instead. As a result, more pro-inflammatory molecules are produced, while apoptosis and necrosis are increased in cardiomyocytes (52, 53). As a consequence, adverse cardiac remodeling and cardiac dysfunction occur (54–57). Furthermore, high MLR has been proven to be associated with an increased risk of cardiac death (9, 58). However, to our knowledge, this is the first study to confirm that high MLR is associated with adverse cardiac remodeling and cardiac dysfunction in elderly patients with AF.

A higher MLR has been reported to be associated with the severity of coronary artery atherosclerotic stenosis (59). However, in our study, an association between MLR and CHD was not found. The association between MLR and the severity of CHD should be explored further in the future.

However, our study is not without its limitations. First, although this study found that the monocyte/lymphocyte ratio was associated with renal dysfunction and adverse cardiac remodeling, as this study was conducted in a single center, there is a possibility of selection bias, and therefore, caution should be exercised when generalizing the findings. Second, the retrospective nature of the study may have resulted in the presence of residual confounding factors, despite our attempts to adjust for possible confounders. The design was retrospective, and important data may be missing. The reasons for the missing data (especially for estimated glomerular filtration rate and plasma albumin) were challenging to determine based on the available information. Inflammatory markers, such as interleukins and cytokines, were not measured in this study. Third, this study had a moderate sample size and selection bias could not be avoided.

## Conclusions

Our study suggested that high MLR is associated with male sex, decreased plasma albumin level, low eGFR, adverse cardiac remodeling, and cardiac dysfunction in elderly patients with AF. High MLR can be used as a marker of subclinical chronic kidney disease and adverse cardiac remodeling among AF patients.

## Data Availability

The original contributions presented in the study are included in the article/Supplementary Material, further inquiries can be directed to the corresponding author.
